# Supplementary material for: A fault-intrusion-tolerant system and deadline-aware algorithm for scheduling scientific workflow in the cloud
Source: PeerJ Comput Sci. 2021 Nov 2;7:e747. doi: 10.7717/peerj-cs.747 (PMC8576554; doi:10.7717/peerj-cs.747)
Supplement: Supplemental Information 6 [file peerj-cs-07-747-s006.zip › Myproject1.1/docs/org/workflowsim/clustering/balancing/methods/class-use/BalancingMethod.html]

Uses of Class org.workflowsim.clustering.balancing.methods.BalancingMethod


JavaScript is disabled on your browser.


- Overview
- Package
- Class
- Use
- Tree
- Deprecated
- Index
- Help

- Prev
- Next

- Frames
- No Frames

- All Classes

## Uses of Class org.workflowsim.clustering.balancing.methods.BalancingMethod

- Packages that use BalancingMethod

  | Package | Description |
  | --- | --- |
  |  |  |
  | --- | --- |
  | org.workflowsim.clustering.balancing.methods |  |
- - ### Uses of BalancingMethod in org.workflowsim.clustering.balancing.methods

    Subclasses of BalancingMethod in org.workflowsim.clustering.balancing.methods

    | Modifier and Type | Class and Description |
    | --- | --- |
    |  |  |
    | --- | --- |
    | `class` | `ChildAwareHorizontalClustering` ChildAwareHorizontalClsutering is a method that merges a task and its children |
    | `class` | `HorizontalDistanceBalancing` HorizontalDistanceBalancing is a method that merges tasks based on distance metric |
    | `class` | `HorizontalImpactBalancing` HorizontalImpactBalancing is a method that merges tasks that have similar impact factors. |
    | `class` | `HorizontalRandomClustering` HorizontalRuntimeBalancing is a method that merges task so as to balance job runtime |
    | `class` | `HorizontalRuntimeBalancing` HorizontalRuntimeBalancing is a method that merges task so as to balance job runtime |
    | `class` | `VerticalBalancing` VerticalBalancing is the same vc as in org.workflowsim.clustering. |

- Overview
- Package
- Class
- Use
- Tree
- Deprecated
- Index
- Help

- Prev
- Next

- Frames
- No Frames

- All Classes
